# Side Effects of Being Blue: Influence of Sad Mood on Visual Statistical Learning

**DOI:** 10.1371/journal.pone.0059832

**Published:** 2013-03-26

**Authors:** Julie Bertels, Catherine Demoulin, Ana Franco, Arnaud Destrebecqz

**Affiliations:** 1 Center for Research in Cognition and Neurosciences (CRCN), Université Libre de Bruxelles (U.L.B.), Brussels, Belgium; 2 Fonds de la Recherche Scientifique – FNRS, Belgium; 3 Fonds National de la Recherche Luxembourg – FNR, Luxemburg; University of Manchester, United Kingdom

## Abstract

It is well established that mood influences many cognitive processes, such as learning and executive functions. Although statistical learning is assumed to be part of our daily life, as mood does, the influence of mood on statistical learning has never been investigated before. In the present study, a sad vs. neutral mood was induced to the participants through the listening of stories while they were exposed to a stream of visual shapes made up of the repeated presentation of four triplets, namely sequences of three shapes presented in a fixed order. Given that the inter-stimulus interval was held constant within and between triplets, the only cues available for triplet segmentation were the transitional probabilities between shapes. Direct and indirect measures of learning taken either immediately or 20 minutes after the exposure/mood induction phase revealed that participants learned the statistical regularities between shapes. Interestingly, although participants from the sad and neutral groups performed similarly in these tasks, subjective measures (confidence judgments taken after each trial) revealed that participants who experienced the sad mood induction showed increased conscious access to their statistical knowledge. These effects were not modulated by the time delay between the exposure/mood induction and the test phases. These results are discussed within the scope of the robustness principle and the influence of negative affects on processing style.

## Introduction

Life is full of events that constantly influence our feelings, resulting in everyone being in a specific mood at specific moments. As mood is part of our daily life, it is crucial to understand how it might interact with cognitive processing. Many studies explored this topic, and the influence of mood on various processes such as learning (e.g., [1]), memory (e.g., [2], [3]) and executive functions (e.g., [4]–[6]) is now well documented. Although the empirical findings are controversial regarding the respective impacts of positive and negative moods on performance, authors usually agree that a positive mood may facilitate a top-down processing style and the use of heuristics (e.g., [7]) while a sad mood may foster analytical and systematical processing, favor bottom-up processes and narrow the attentional focus by increasing attention to detail [7]–[9].

In particular, according to Schwarz [7]’s feelings-as-information theory, our affective states inform us about the nature of the situation we are in. Consequently, negative emotions enlighten that action is required in order to get more positive outcomes and avoid negative ones, while positive emotions do not require any specific action to be taken. These differences in terms of action requirements may lead to divergences in information processing under the influence of positive or negative affective states. Specifically, Schwarz assumed that a negative affective state would lead to (1) focusing attention on the features of the affect-eliciting event, and to (2) causal reasoning about this event. In other words, negative affective states would favor analytic processing strategies.

The impact of affective states might generalize to on-going unrelated cognitive tasks. According to Schwarz [7], the narrower focus of attention elicited by negative events would result in information being categorized more narrowly and stored in smaller chunks. Also, the propensity of causal, analytical reasoning of individuals in negative moods would result in more accurate contingency assessments and facilitated covariation detection as compared to elated individuals.

Although the influence of affective states on implicit processes such as stereotype activation or semantic priming has been widely studied (e.g., [10], [11]), few authors investigated how mood might interfere with implicit learning, namely the incidental acquisition of complex knowledge. This is probably related to the considerable support received by the ‘robustness principle’ (e.g., [12]) in the related field of implicit memory research. This principle, developed within the context of evolutionary biology, states that implicit processes, antedating explicit ones, should be more robust in the face of various disorders and dysfunctions, as well as regarding individual differences.

Nevertheless, recent studies reported higher performance on implicit learning tasks such as the detection of covariation and artificial grammar learning, in participants in whom a sad (vs. happy or neutral) mood was induced [13], [14]. These results, however, do not unequivocally support an influence of mood on implicit learning, as there are good reasons to believe that none of these tasks can be considered as process-pure, namely as involving exclusively implicit, but no explicit knowledge. Indeed, previous studies have shown that explicit knowledge also contributes to performance in the detection of visual covariation [15] and in artificial grammar learning [16]. Further studies are therefore needed to ascertain whether a sad mood induction increases conscious or unconscious knowledge acquisition under incidental learning instructions.

The present study aimed at investigating whether and to what extent mood impacts on visual statistical learning. Statistical learning (SL) refers to the ability to discover systematic patterns embedded in a continuous stream of auditory or visual stimuli. Given the complexity of our sensorial environment, SL is considered as a fundamental aspect of human cognition. Still, to our best knowledge, no study so far investigated the potential influence of affective states on SL.

SL is generally considered as unintentional and automatic. Therefore, it is often viewed as a form of incidental learning very similar to implicit learning [17]. However, even though the extracted regularities cannot be easily verbalized, the implicit nature of statistical learning is a matter of debate. Indeed, a recent study demonstrated that visual SL could not be completely accounted for by implicit knowledge acquisition [18].

Here, our purpose is to examine to what extent the induction of a sad mood influences learning of the statistical regularities present in a sequence of visual shapes and/or the implicit or explicit nature of the acquired representations.

To this aim, we presented a sad vs. neutral story during exposure to the visual shapes in order to induce a sad vs. neutral mood to the participants. Although mood is usually induced before the experiment, we chose to combine the induction and the exposure phases because we specifically wanted to investigate the effect of mood on the acquisition of statistical regularities. Moreover, as mood induction is known to be relatively transient, this procedure increases the probability that participants will still be experiencing the induced mood in the last part of our 10 minutes exposure phase. Mood questionnaires were filled in before and after the mood induction in order to support the effectiveness of the mood change.

As in Bertels et al. [18], participants’ knowledge of the statistical regularities was then assessed through indirect and direct measures of learning (a rapid serial visual presentation (RSVP) and a four-choice completion tasks, respectively). Half of the participants performed these tasks immediately after the exposure/mood induction phase, while the other half performed them after a 20 minutes delay. Indeed, unpublished data from our laboratory show the effects of mood induction usually vanish after 20 minutes, while recent studies have shown that visual statistical learning is lasting and consistent over time [19], [20]. Hence, participants in the delayed condition should have recovered from any mood induction when starting the RSVP and the four-choice completion tasks. Participants in the delayed condition were also asked to fill in the mood questionnaires for a third time at the end of the 20 minutes break. This aimed at ruling out the possibility that any effect observed in the RSVP and the four-choice completion tasks would be due to mood-related influences at test rather than on the learning process itself.

We also used subjective measures of performance, namely binary confidence judgments taken after each completion trial, in order to measure awareness. Combining subjective and objective measures would allow us to clarify the explicit or implicit nature of the acquired knowledge, and to assess the influence of mood on awareness.

We expect to replicate the results of our previous study [18], namely to observe that participants learn the statistical regularities. According to Schwarz [7], a negative mood elicits a restricted focus of attention and results in information being categorized more narrowly and stored in smaller chunks. A negative mood would also produce a higher degree of causal, analytical reasoning resulting in more accurate contingency assessments and covariation detection. Based on these claims, we predict that visual SL would be improved in the sad mood induction group as compared to the neutral group. This would lead to better performance in the completion task and more explicit knowledge of the sequences in ‘sad’ than ‘neutral’ participants. The robustness principle [12] would rather predict that both groups would not differ regarding implicit visual SL.

## Method

### Participants

Participants were 128 students of the Université Libre de Bruxelles (93 women), ranging from 18 to 44 years (mean: 21.45). They received course credits for their participation. All reported (corrected-to-) normal vision. Participants were randomly assigned to one of the four experimental conditions (Neutral vs. Sad mood and ‘Delay 0′ vs. ‘Delay 20′ condition).

### Ethics statement

This experiment was a mandatory component of a practical course in psychology. Participants received course credits for their participation. All participants gave their oral consent to participate in the study. They were informed that they would be exposed to a sad or neutral story and would be asked to perform several tasks on a computer. They would also have to anonymously fulfill several questionnaires which questions might be intrusive. Participants were informed that they could withdraw from the experiment at any point. No particular written consent form was necessary, as they already provided a general consent for the course to the Psychology Faculty. The study and the verbal consent procedure were approved by the Ethics Committee of the Psychological and Educational Sciences Faculty of the Université Libre de Bruxelles.

## Materials and Apparatus

### Mood Induction Procedure (MIP)

To induce mood, participants listened to a story told by an actress played on an IPod. They were asked to pay attention to the story, with no further recommendation. In the neutral mood group, the story was supposedly neutral, and consisted in the biography of Nicolas Leonard Sadi Carnot, French physicist and engineer from the 19^th^ century [21]. In the sad mood group, the story was an excerpt of a novel by Henry Bauchau [22], and is about a young mother in the terminal phase of cancer.

In order to support that our stories indeed differed in terms of emotional valence but not in terms of arousal, 64 participants (from the ‘Delay 20′ condition) were asked to draw a mark on two visual analogue scales (VAS) that fitted the best their judgment about the content of the story. They were asked to respond independently from their current personal feeling and emotions. These lines referred to how negative/positive and how calming/arousing they found the story was. Also, in order to ascertain that the sad story was indeed sadder than the neutral one, and that both stories did not differ in terms of interest, participants had to rate on seven-points scales ranging from (1) “not at all” to (7) “totally”, to what extent the adjectives “sad” and “interesting” described the story they just listened to. One-way ANOVAs revealed that both stories differed in terms of valence, with the sad story (M = 3.4938, SD = 2.081) judged as more negative than the neutral story (M = 4.722, SD = 2.055), F(1,63) = 5.642, p = .021, but not in terms of arousal (M = 4.125, SD = 1.802 and M = 3.428, SD = 2.447, respectively), F(1,63) = 1.683, p = .199. Also, the sad story was judged significantly sadder than the neutral one (M = 5.9687, SD = 1.356 vs. M = 1.625, SD = .976), F(1,63) = 216.462, p<.001. The two stories did not differ in terms of interest (M = 3.813, SD = 1.554 and M = 3.594, SD = 1.72), F <1.

Before and after the MIP, participants completed the French version of the Brief Mood Introspection Scale (BMIS) ([23], translated by [24], see e.g., [25]) and the affect grid [26]. The BMIS is a 16-item self-report questionnaire in which each adjective, denoting a positive or negative trait, is rated on a 4-point scale. The affect grid consists in a matrix of 81 squares (9 lines × 9 columns), with the horizontal and vertical dimensions of the matrix representing the degrees of valence and arousal, respectively. The participants were asked to mark a square in the matrix that represents the best their current affective state.

### Learning tasks

**Figure 1:**
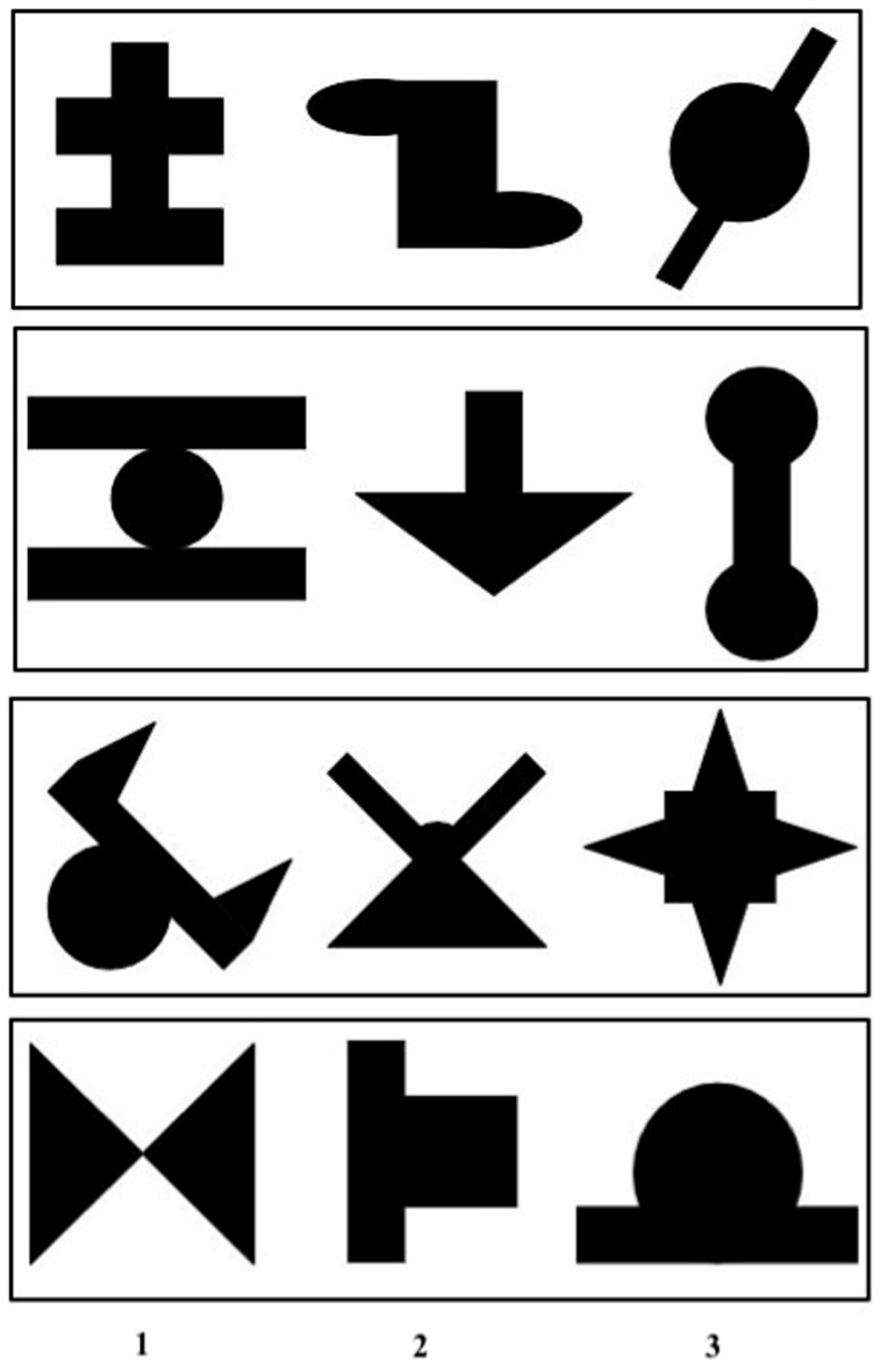
Triplets. Groups of three shapes constituting each of the four triplets, by order of presentation (1, 2, 3).

Visual stimuli consisted in 12 black shapes presented on a white background, adapted from Fiser and Aslin [27]. They were about 3 cm long and 3 cm high. These stimuli were combined to form four ‘triplets’, namely four sequences of three stimuli presented in a fixed order (Figure 1).

Stimulus presentation, timing and data collection were controlled using the Psyscope USB button box and Psyscope X software [28] running on a Mac mini 1.33 GHz PowerPC G4.

## Procedure

### Exposure/Mood Induction Phase

Right before exposure, participants filled in the BMIS [23] and the affect grid [26]. Then, the experimenter started the IPod together with the exposure phase.

Exposure was composed of 1230 trials, consisting of 100 repetitions of each of the twelve shapes forming the triplets plus 30 trials consisting in three presentations of 10 black letters shown on a white background. Stimuli were presented one at a time, for 250 ms, with a 250 ms inter-stimulus interval. Each of the twelve shapes was presented in the fixed order defined by the stimulus make-up and the triplet it was part of. Participants were not told about these sequential regularities. Four exposure sequences were generated, in which triplets were pseudo-randomly presented: a given triplet was never presented twice in a row. The presentation of the shapes was randomly interspersed with the presentation of the letters. Participants were asked to detect the letters by pressing the right key. This procedure was used to ensure that participants paid attention to the stimuli presented in the exposure phase without explicitly making them aware of the sequence of shapes. These data were not considered in the analyses.

Both exposure and the story lasted about 10 minutes, after which participants filled in the BMIS [23] and the affect grid [26] for the second time.

### Delay 0/Delay 20

**Figure 2:**
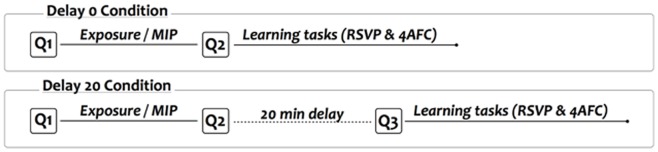
Delay. Time-course of ‘Delay 0′ and ‘Delay 20′ conditions.

In the ‘Delay 0′ condition, participants started the Rapid Serial Visual Presentation (RSVP) task right after having filled the mood questionnaires for the second time. In the ‘Delay 20′ condition, there was a 20-minutes break between the Exposure/MIP phase and the RSVP task. As mentioned in the Introduction, the aim of this time delay was to allow participants to return to a baseline mood (i.e., a similar mood as before starting the experiment). Unpublished data from our lab indeed showed that a 20 minutes break was long enough to recover from a MIP. During this break, participants had to assess the emotional valence, the arousal, the sadness and the interest of the story they listened to (see Material and Apparatus section), and to fill in questionnaires about their sleep environment, their videogames habits, their musical skills and their linguistic expertise. In case they filled in these questionnaires in less than 20 minutes, participants played Pong, one of the earliest arcade videogame simulating a tennis sports game. After this break, participants in the ‘Delay 20′ condition filled in the mood questionnaires for the third time, right before starting the RSVP task. Figure 2 displays the time course of both conditions.

### RSVP task

The first test consisted in a rapid serial visual presentation paradigm in which participants had to detect a target within a stream of stimuli. On each trial, the presentation of the target (one of the twelve shapes presented during Exposure) was followed by the presentation of the four triplets, one shape at a time, at the same rate as during Exposure. Participants were asked to press the right key as soon as they saw the target. The RSVP stream was then interrupted, and the next target was presented. Each target shape was presented six times, in the first, second or third position of the second or third triplet in the RSVP stream, resulting in 72 trials. The presentation order of the triplets was counterbalanced across participants.

The rationale was that if participants learned the statistical regularities of the triplets during the exposure phase, reaction times (RTs) should be faster to the second and third predictable elements of each triplet than to the first, unpredictable element.

### Completion task

The second test consisted in a four alternatives forced choice (4 AFC) task in which participants were presented with a triplet in which one shape was missing. They had to pick one shape among four presented underneath to complete the triplet. These shapes were part of the triplets presented before, and their position corresponded to the position of the missing shape in the to-be-completed triplet. Participants responded by pressing one of four keys. Each triplet was presented six times, resulting in 24 trials. The same missing shape in the first, second or third location was thus presented twice, with a different presentation order of the four possible shapes. The sequence of completion trials was counterbalanced across participants.

After each trial, participants gave a binary confidence judgment regarding their completion response. Namely they had to indicate whether they guessed (i.e., they had no idea whatsoever concerning the correct response, their answer was as good as flipping a coin) or remembered (i.e., they felt that their response was based on some recollection of the learning material) by pressing one of two keys (for similar labels, see e.g. Dienes & Seth [29]).

## Results

### Mood Induction Procedure

**Efficiency of the MIP (n = 128):** Four repeated measures analyses of variance (ANOVA) were applied to participants’ scores on (1) the positive items of the BMIS, (2) the negative items of the BMIS, (3) the valence scale of the affect grid, and (4) the arousal scale of the affect grid. Separate analyses were made for the subscales of each questionnaire rather than considering the subscale as a within-subject factor. Indeed, there is no interest in directly comparing the scores at the different subscales of a questionnaire (e.g., in the affect grid, it is irrelevant to compare the scores on the valence and on the arousal dimensions). Moreover, in the BMIS the average scores for the positive and negative items are computed on a different number of items (9 vs. 7 items, respectively). In each ANOVA, Mood was a between-subjects factor (2 levels: neutral, sad) and Moment of Assessment (2 levels: Pre-MIP, Post-MIP) was a within-subject factor. The factor Delay (i.e. whether participants had a break between the Exposure/MIP phase and the learning tasks) was considered irrelevant for these analyses given that, at this stage, the course of the experiment was the same in all participants (see Figure 2). Table 1 displays the average raw scores.

**Table 1:** Efficiency of the MIP.

	BMIS		BMIS		AffectGrid		AffectGrid	
	Positive Items		Negative Items		Valence Scale		Arousal Scale	
	Pre-MIP	Post-MIP	Pre-MIP	Post-MIP	Pre-MIP	Post-MIP	Pre-MIP	Post-MIP
Neutral MIP	24.719	22.625	23.609	23.563	1.127	.984	.476	.127
	(.528)	(.602)	(.425)	(.415)	(.166)	(.197)	(.178)	(.183)
Sad MIP	24.484	21.781	23.5	21.984	1.344	.141	.375	−.438
	(.528)	(.602)	(.425)	(.415)	(.165)	(.195)	(.177)	(.181)
Average	24.602	22.203	23.555	22.773	1.235	.562	.426	−.155
	(.373)	(.426)	(.3)	(.293)	(.117)	(.139)	(.126)	(.129)

Average raw scores obtained on the BMIS positive and negative items and on the Affect Grid valence and arousal scales (all participants, n = 128). Standard deviations are in parentheses.

The first ANOVA conducted on the scores on positive items of the BMIS revealed a significant effect of Moment, F(1,126) = 69.298, p<.001, partial η^2^ = .355, indicating that participants were less positive immediately after than before the MIP. This effect did not interact with Mood, F(1,126) = 1.118, p = .292. The effect of Mood was not significant either, F <1. This is most probably due to the experimental situation.

The second ANOVA on the scores on negative items of the BMIS revealed a significant effect of Moment, F(1, 126) = 9.796, p = .002, partial η^2^ = .072, indicating that participants were more negative immediately after than before the MIP. The Mood x Moment interaction was also significant, F(1,126) = 8.656, p = .004, partial η^2^ = .064. As predicted, Bonferroni adjusted comparisons revealed that ‘sad’ participants reported a more negative mood after than before the MIP, p<.001. This was not the case for ‘neutral’ participants’ scores, which did not differ before and after the MIP, p = .895. Also, while after the MIP participants in the sad condition reported to be in a significantly more negative mood than participants in the neutral mood condition, p = .008, scores did not differ between mood conditions before the MIP, p = .856. The main effect of Mood was not significant, F(1,126) = 2.456, p = .120.

The third ANOVA on the scores on the affect grid valence scale (made on the data of 127 participants since one of them forgot to fill the affect grid both before and after the MIP) revealed a significant effect of Moment, F(1,125) = 37.825, p<.001, partial η^2^ = .232, and a significant Mood x Moment interaction, F(1,125) = 23.471, p<.001, partial η^2^ = .158. As for the BMIS and coherently with our predictions, Bonferroni adjusted comparisons revealed that although ‘neutral’ participants’ scores did not differ before and after the MIP, p = .36, ‘sad’ participants reported a more negative mood after than before the MIP, p<.001. Moreover, while scores did not differ between mood conditions before the MIP, p = .356, after the MIP participants in the sad condition reported to be in a significantly more negative mood than participants in the neutral mood condition, p = .003. The effect of Mood was not significant, F(1,125) = 1.825, p = .179.

The fourth ANOVA on scores on the affect grid arousal scale (made on the data of 127 participants, see before) revealed a significant effect of Moment, F(1,125) = 27.885, p<.001, partial η^2^ = .182, and a significant Mood x Moment interaction, F(1,125) = 4.435, p = .037, partial η^2^ = .034. Bonferroni adjusted comparisons revealed that while scores did not differ between mood conditions before the MIP, *p* = .688, after the MIP participants in the sad condition reported to be significantly less aroused than participants in the neutral mood condition, p = .03. Both ‘neutral’ and ‘sad’ participants were less aroused after than before the MIP, p = .027 and p<.001. This is most probably due to the calming effect of listening to a story, combined to the effect of the sad nature of the sad story.

### Efficiency of a 20 minutes delay in recovering a baseline mood in the ‘Delay 20′ condition (n = 64)

As in the previous section, four repeated measures ANOVAs were applied on participants’ scores. Mood was a between-subjects factor and Moment of Assessment (3 levels: Pre-MIP, Post-MIP, 20′ Post-MIP) was a within-subject factor. Due to a technical problem, the post-MIP data of one 'Delay 20' participant were lost. The analyses have been made on the remaining 63 participants. Table 2 displays the average raw scores.

**Table 2:** Efficiency of a 20 minutes delay in recovering a baseline mood in the ‘Delay 20′ condition (n = 64).

	BMIS Positive Items			BMIS Negative Items			Affect Grid Valence Scale			Affect Grid Arousal Scale		
	Pre-MIP	Post-MIP	20' Post-MIP	Pre-MIP	Post-MIP	20' Post-MIP	Pre-MIP	Post-MIP	20' Post-MIP	Pre-MIP	Post-MIP	20' Post-MIP
Neutral MIP	25.438	23.125	23.844	24.563	24.281	25.375	1.406	1.313	1.719	.500	.125	.687
	(.68)	(.803)	(.717)	(.357)	(.513)	(.43)	(.215)	(.26)	(.231)	(.252)	(.239)	(.257)
Sad MIP	25.258	22.194	24	25.226	23.032	24.677	1.806	.387	1.581	.710	−.452	1.29
	(.691)	(.816)	(.728)	(.363)	(.521)	(.436)	(.219)	(.264)	(.235)	(.256)	(.242)	(.261)
Average	25.348	22.659	23.922	24.894	23.657	25.026	1.606	.85	1.65	.605	−.163	.989
	(.484)	(.572)	(.511)	(.255)	(.366)	(.306)	(.154)	(.186)	(.165)	(.18)	(.17)	(.183)

Average raw scores obtained on the BMIS positive and negative items and on the Affect Grid valence and arousal scales (participants in the ‘Delay 20′ condition, n = 64). Standard deviations are in parentheses.

The first ANOVA conducted on scores on the positive items of the BMIS revealed a significant main effect of Moment, F(2,122) = 20.67, p<.001, partial η^2^ = .253. Bonferroni adjusted comparisons revealed that participants were more positive 20 minutes after than immediately after the MIP, p = .017, but less positive 20 minutes after than before the MIP, p = .003. Again, this is most probably due to the experimental situation. This effect did not interact with Mood, F <1. The effect of Mood was not significant either, F <1.

The second ANOVA on the scores on the negative items of the BMIS revealed a significant effect of Moment, F(2,122) = 13.017, p<.001, partial η^2^ = .177, and a significant Mood x Moment interaction, F(2,122) = 5.563, p = .005, partial η^2^ = .084. Considering mood conditions separately, we found the effect of Moment to be significant in both neutral, F(2,62) = 4.593, p = .014, partial η^2^ = .129 and sad groups F(2,60) = 12.417, p<.001, partial η^2^ = .293. In the neutral group, Bonferroni adjusted comparisons revealed that participants were less negative after 20 minutes than immediately after the MIP, p = .005. There was no significant difference on scores on the negative items of the BMIS before the MIP and 20 minutes after, p = .199. In the sad group, Bonferroni adjusted comparisons revealed that, coherently with our predictions, participants were less negative after 20 minutes than immediately after the MIP, p = .009, while there was no difference before and 20 minutes after the MIP, p = .643.

The third ANOVA on scores on the affect grid valence scale revealed a significant effect of Moment, F(2,122) = 14.897, p<.001, partial η^2^ = .196, and a significant Mood x Moment interaction, F(2,122) = 8.181, p<.001, partial η^2^ = .118. Considering mood conditions separately, we found the effect of Moment to be significant in the sad group, F(2,60) = 17.101, p<.001, partial η^2^ = .363, but not in the neutral group, F(2,62) = 2.184, p = .121. Bonferroni adjusted comparisons revealed that, coherently with our predictions and the previous analysis, participants in the sad mood group were less negative 20 minutes after than immediately after the MIP, p = .001, while there was no difference before and 20 minutes after the MIP, p = .913. The effect of Mood was not significant, F <1.

The fourth ANOVA on scores on the affect grid arousal scale revealed a significant effect of Moment, F(2,122) = 20.453, p<.001, partial η^2^ = .251, and a significant Mood x Moment interaction, F(2,122) = 5.358, p = .006, partial η^2^ = .081. Considering mood conditions separately, we found the effect of Moment to be significant in the sad group, F(2,60) = 21.343, p<.001, partial η^2^ = .416, but not in the neutral group, F(2,62) = 2.679, p = .077. Bonferroni adjusted comparisons revealed that participants in the sad mood group were more aroused 20 minutes after than immediately after the MIP, p<.001.This effect did not reach significance when comparing the scores 20 minutes after and before the MIP, p = .059. These results support the idea that participants recovered from the induced sad mood that reduced their arousal level immediately after the story (see previous section). The effect of Mood was not significant, F <1.

## Learning tasks

### Overall Analyses

#### RSVP task

As misses and erroneous detections were rare (4.254% of the trials), analyses were performed only on correct RTs.

**Figure 3:**
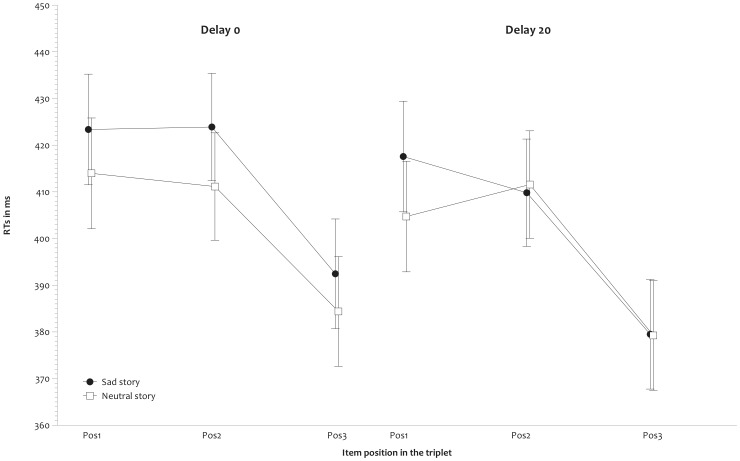
RTs in the RSVP task. Mean detection latencies for the three positions (Pos1, Pos2, Pos3) in the RSVP task, plotted separately for the neutral and sad mood groups, and by Delay condition.

A repeated measures ANOVA was applied on response latencies, with Mood (2 levels: neutral, sad) and Delay (2 levels: delay 0, delay 20) as between-subjects factors and Position (3 levels: 1, 2, 3) as a within-subject factor. This analysis revealed a significant effect of Position, F(2,248) = 144.887, p<.001, partial η^2^ = .539. Bonferroni adjusted comparisons revealed that RTs in Position 3 (384 ms) were significantly faster than in Positions 1 and 2 (415 and 414 ms), both p<.001, which did not differ from each other, F <1. These results indicate that, on average, participants learned the triplets. Neither the effect of Mood nor its interaction with Position or Delay were significant, F(1,124) = 1.528, p = .219, F(2,248) = 1.581, p = .208 and F <1 (see Figure 3). The triple Mood x Position x Delay interaction did not reach significance, F(2,248) = 2.392, p = .094.

#### Completion task

The overall completion performance was 31.664% (SD = 13.271%) in the neutral group and 31.768% (SD = 14.709%) in the sad group. Both differed significantly from a chance-level of 25%, t(63) = 4.005, p<.001, Cohen’s d = 1.009 and t(63) = 3.681, p<.001, Cohen’s d = .928. An univariate ANOVA with Mood and Delay as fixed factors revealed that these scores did not differ as a function of Mood or Delay, F <1 and F(1,124) = 2.337, p = .129, respectively. The Mood x Delay interaction did not reach significance, F <1. These results indicate that participants learned the triplets similarly in all groups (see Table 3).

**Table 3:** Performance and confidence in the completion task.

	All participants				Participants above chance in the 4 AFC task			
	(n = 128)				(n = 70)			
	Performance		Confidence		Performance		Confidence	
	Delay 0	Delay 20	Delay 0	Delay 20	Delay 0	Delay 20	Delay 0	Delay 20
Neutral MIP	29 (2)	34 (2)	24 (5)	37 (5)	36 (3)	42 (3)	29 (6)	43 (6)
Sad MIP	31 (2)	33 (2)	37 (5)	33 (5)	42 (3)	44 (3)	48 (7)	38 (6)

Average performance and confidence in the 4 AFC task, for all participants and for participants that performed above chance level. Standard deviations are in parentheses.

Overall, ‘neutral’ participants judged that they remembered which was the missing shape in 30.468% of the cases (SD = 27.287%) and that they guessed in the remaining 69.532%. In the sad group, participants’ confidence (i.e., the percentage of “remember” responses) raised to 34.898% (SD = 25.323%). An univariate ANOVA with Mood and Delay as fixed factors revealed that these scores did not differ as a function of Mood or as a function of Delay, F <1 and F(1,124) = 1.094, p = .298, respectively. The Mood x Delay interaction approached significance, F(1,124) = 3.592, p = .06, partial *η2* = .028 (see Table 3). Considering both delay conditions separately, we found that ‘sad’ participants were more confident in their responses than ‘neutral’ ones when the RSVP and completion tasks immediately followed the MIP, F(1,63) = 4.551, p = . 037, partial *η2* = .068, but not when there was a 20-minutes break, F <1. Moreover, ‘neutral’ participants were more confident in the Delay 20 than in the Delay 0 condition, F(1,63) = 4.137, p = .046, partial *η2* = .063. No such difference was observed in ‘sad’ participants, F <1.

Although, on average, participants performed above chance, about half of them (n = 58) were actually at chance in the completion task, obtaining only 25% or less of correct responses. These participants were significantly less confident in their responses than participants performing above chance level (24.498%, SD = 24.879 vs. 39.465%, SD = 25.692), F(1,127) = 11.077, p = .001, Cohen’s d = .592. The proportion of participants at chance did not differ between groups (n = 25 in the neutral and n = 33 in the sad group, χ^2^(1, N = 128) = 2.018, p = .155) or between delay conditions (n = 31 in the Delay 0 and n = 27 in Delay 20 condition, χ^2^(1, N = 128) = .504, p = .478). These results are in line with the fact that performance in the completion task did not differ between both mood and delay conditions.

Results from participants who performed above or at chance in the completion task are considered separately in the following analyses (for a similar procedure, see Bertels et al. [18]).

Focusing on participants who performed above chance, we investigated whether their knowledge was above the subjective criterion of consciousness, namely whether they had some metaknowledge about their statistical knowledge. To this aim, we used two indicators: the zero correlation criterion [30] and the guessing criterion [31]. The zero-correlation criterion is met when there is no relationship between confidence levels and performance rates. In other words, if participants are not aware of their knowledge, high and low confidence ratings should be randomly assigned to correct and incorrect discriminations. Conversely, if performance is based on conscious knowledge, participants should be more confident in their correct responses than in their errors [30]. Confidence judgments can be further used to implement the Signal Detection Theory (SDT), in which correct discriminations made with high confidence are considered as Hits and incorrect discriminations made with high confidence as False Alarms [32]. This procedure would result in Type II d’ (representing participants’ awareness of their own performance) reliably above zero when participants have conscious access to their knowledge. The main advantage of the SDT procedure in this context is to provide an unbiased measure of awareness, unaffected by participants’ own report criterion for making high and low confidence judgments ([32], [33]; but see [34]).

According to the guessing criterion, knowledge is below the subjective threshold of consciousness when performance is above chance while participants claim to guess.

Regarding participants who performed at chance in the direct task, we investigated whether they actually learned the triplets during exposure based on their results in the indirect task.

### Participants who performed above chance in the completion task (n = 70)

Completion performance reached 39.426% of correct responses (SD = 10.76%) in the neutral group (n = 39) and 42.742% (SD = 13.816%) in the sad group (n = 31). ‘Neutral’ participants who performed above chance reported to remember the missing shape in 36.752% of the cases (SD = 27.621%), while ‘sad’ participants who performed above chance reported to remember it in 42.878% of the cases (SD = 23.03%, see Table 3).

**Figure 4:**
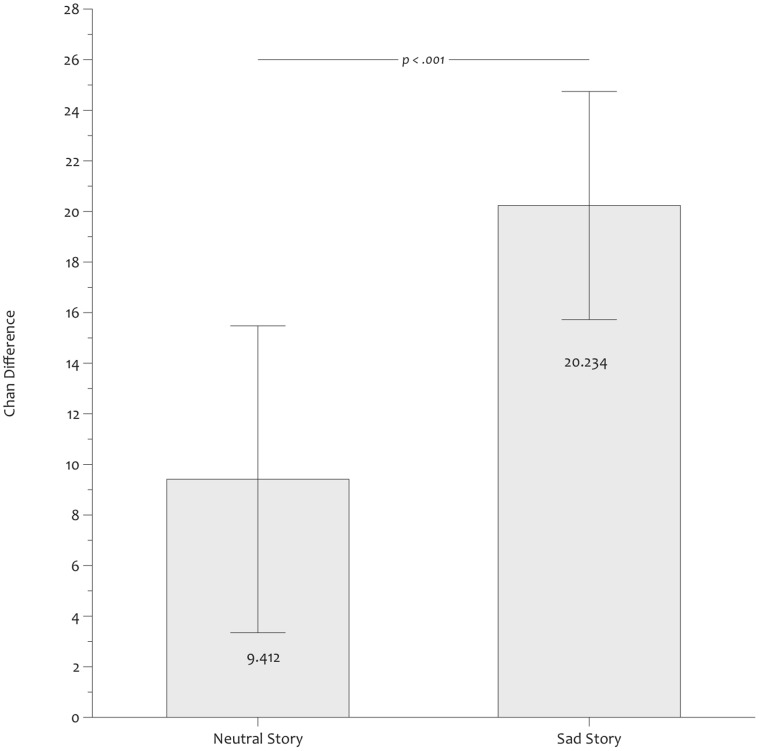
Chan differences. Differences between the proportions of “remember” responses in correct and incorrect completions in participants who performed above chance level in the completion task, represented separately for sad and neutral mood groups.

We observed that these participants who performed above chance made significantly more “remember” judgments when they were correct than incorrect, both in the neutral (Chan difference = 9.412, SD = 18.714) and in the sad mood groups (Chan difference = 20.234, SD = 19.528), t(38) = 3.141, p = .003 and t(30) = 5.799, p<.001, respectively (see Figure 4). These results indicate conscious knowledge by the zero correlation criterion in both mood groups. In line with our predictions, an univariate ANOVA with Mood and Delay as fixed factors revealed that these Chan differences differed as a function of Mood, F(1,66) = 5.484, p = .022, partial η^2^ = .077. Neither the effect of Delay nor the Mood x Delay interaction were significant, both F <1.

We also computed Type II d’ where a “remember” judgment for a correct response is considered as a Hit and a “remember” judgment for an error as a False Alarm. In line with the previous results, we observed that Type II d’ in the neutral (.28, SD = .746) and in the sad groups (.935, SD = 1.042) differed significantly from zero, t(38) = 2.347, p = .024, Cohen’s d = .761 and t(30) = 4.997, p<.001, Cohen’s d = 1.825, respectively. Also, an univariate ANOVA revealed that Type II d’ differed as a function of Mood, F(1,66) = 9.506, p = .003, partial η^2^ = .126, but neither the effect of Delay nor the Mood x Delay interaction reached significance, both F <1.

**Figure 5:**
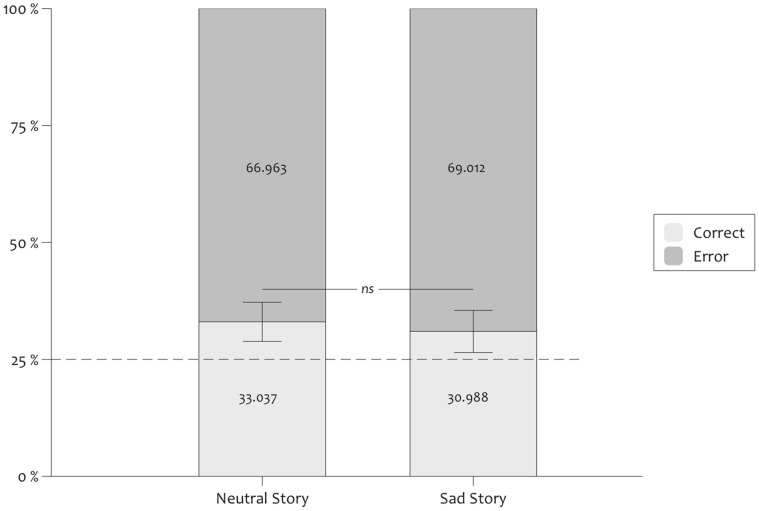
Guessing criteria. Proportion of correct completions when participants who performed above chance level in the completion task claimed to guess, represented separately for sad and neutral mood groups.

Importantly, ‘neutral’ participants who performed above chance in the completion task did so even when they claimed to guess the correct shape (in 33.037% of the cases, SD = 12.932%, t(38) = 3.881, p<.001, Cohen’s d = 1.259, Figure 5). This was also the case for ‘sad’ participants (30.988%, SD = 12.292%, t(30) = 2.712, p = .011, Cohen’s d = .99). An univariate ANOVA with Mood and Delay as fixed factors revealed that these scores did not differ as a function of Mood or as a function of Delay, both F <1. The Mood x Delay interaction was also not significant, F <1. According to the guessing criterion, such results suggest that completion performance in both mood groups was at least partly based on unconscious knowledge.

### Participants who performed at chance in the completion task (n = 58)

A repeated measures ANOVA with Mood and Delay as between-subjects factors and Position as a within-subject factor revealed that RTs differed between target positions, F(2,108) = 63.385, p<.001, partial η^2^ = .54. RTs were faster in Position 3 than in Positions 1 and 2 (385 vs. 416 and 413 ms, respectively), both p<.001. Neither the effect of Mood nor its interaction with Position or Delay were significant, F <1, F(2,108) = 1.483, p = .231 and F <1, respectively. The triple Mood x Position x Delay interaction was not significant, F <1.

The dissociation between the direct and the indirect measures of learning supports the notion that participants who performed at chance in the completion task nevertheless acquired statistical knowledge that cannot be used in the direct task.

## Discussion

The present study aimed at investigating the influence of mood on visual SL. For this purpose, a sad vs. neutral mood was induced in participants during their exposition to sequences of visual shapes. Learning of the sequences was then assessed using indirect and direct tasks, either immediately or 20 minutes after the exposure/mood induction phase. Participants’ confidence in their responses was also collected.

First of all, it is important to note that our mood induction procedure was effective. As a matter of fact, according to the negative items of the BMIS and the valence scale of the affect grid, while there was no difference between groups at the beginning of the experiment, participants in the sad but not in the neutral group reported to be in a more negative mood immediately after the MIP than before. Moreover, the 20 minutes delay between the end of the MIP and the beginning of the learning tasks was successful in recovering a baseline mood. Indeed, according to the negative items of the BMIS and the valence scale of the affect grid, participants reported a less negative mood 20 minutes rather than immediately after the MIP. After 20 minutes, participants’ mood was similar as it was before the MIP.

Both direct and indirect tasks revealed that participants learned the triplets. As a matter of fact, RTs in the RSVP task were modulated by the strength of the transitional probabilities between shapes. In addition, most participants were able to reproduce, at least partly, the training sequences in the 4 AFC completion task. Interestingly, the combined use of objective and subjective measures of performance revealed that participants’ performance cannot be exclusively accounted for by implicit knowledge. Rather, performance was associated with subjective confidence in participants who performed above chance in the completion task, indicating that at least part of their knowledge was explicit. Our results are therefore in line with a previous report indicating that visual SL was mostly based on explicit knowledge acquisition [18]. Coherently with recent studies arguing that statistical learning is lasting and consistent over time [19], [20], these results did not differ according to whether the test phase immediately followed exposure phase or occurred after a 20 minutes break.

We did not observe any quantitative dissociation between sad and neutral groups, either in the direct or in the indirect tasks, and whatever these tasks were performed immediately or 20 minutes after the exposure/mood induction phase. However, the use of subjective measures allowed us to show a qualitative dissociation between both groups regarding participants’ ability to identify the nature of the knowledge they use in the completion task. Specifically, we observed that performance in the completion task was more related to confidence in participants who experienced the sad mood induction. Explicit learning would then be larger in those ‘sad’ participants than in those who listened to the neutral story. That effect did not interact with the Delay condition. Because previous studies have demonstrated that the processing of emotional stimuli requires some level of attention such that the effects of emotional stimuli only occur when sufficient resources are available [34], [35], [36], we checked for the possibility that explicit learning in the sad group came at the expense of attending to the sad story. This was done by assessing the effectiveness of the MIP (considering the scores on the negative items of the BMIS and the scores on the valence scale of the Affect Grid) for participants who performed above chance level in the 4 AFC task and who had a Chan difference above zero (n = 49). Interestingly, both scales revealed that participants’ mood was significantly more negative after than before the MIP in the sad group, p = .019 and p<.001. This was not the case in the neutral group, both p>.10. Importantly, the effectiveness of the MIP in the sad mood group (either considering scores on the BMIS or on the Affect Grid) did not differ according to whether ‘sad’ participants had conscious knowledge or not, *F* <1.

By contrast, the proportion of correct responses when participants reported to guess (i.e., the guessing criterion, reflecting the knowledge about which participants lack metaknowledge) did not differ as a function of mood. These results are thus in line with Reber [12]’s controversial robustness principle that individual differences have less impact on implicit than explicit processes. These results contribute to the substantial body of evidence showing that implicit learning shows greater robustness than explicit learning.

Such a dissociation is consistent with previous studies reporting that negative affective states and emotional disorders affect explicit but not implicit processes [38]–[40], and that an induced sad mood exerts a stronger influence on learning in tasks more likely to recruit explicit than implicit processes, such as the artificial grammar learning vs. the serial reaction time task [14].

Why is conscious access to statistical knowledge increased in participants from the sad mood group? A potential explanation might be the narrow focus of attention and the analytic reasoning style elicited by negative affects and resulting in accurate contingency appraisals [7]. Such a processing style may not only induce participants to be particularly attentive to the shapes rather than to the stream as a whole, but also to their temporal binding [7]. Interestingly, the task itself involved sustained attentional processing as it consists in carefully attending the stream of shapes in order to detect interspersed letters. According to Forgas [41], the effect of negative mood should be even larger if the task characteristics and the participant’s mood state favor the same systematic and detail-oriented processing strategy.

This explanation based on Schwarz [7] might seem at odds with more recent versions of the affect-as-information theory (see e.g., [42]–[44]) stating that while positive affect would promote a relational processing, negative affect would rather inhibit such processing style and favor an item-specific processing. Learning transitional probabilities between shapes within a triplet may indeed be considered as being more relational than item-specific. It should be noted however that relational processing may be considered as a top-down and expectation-driven while item-specific processing is bottom-up and data-driven [44]. In the memory domain, relational processing would refer to connecting semantically or conceptually related items either stored in memory or displayed during the task (see [45]). Yet, in the present study, participants rather focus on shapes presented one at a time and in an item-specific, analytic perspective. Participants may incidentally find contingencies between subsequently presented items but the analytic, causal processing style promoted by negative affective states (as proposed by Schwarz [7]) would then be item-specific rather than relational.

While this analytic processing style promoted awareness of the regularities, it did not enhance performance in the 4 AFC task. One reason may be that the Mood/Exposure phase was performed under dual task condition in which participants had to pay attention to the story while detecting the letters in the stream. This latter task was not difficult per se but demanding in terms of cognitive resources and may have limited performance improvement in the subsequent 4 AFC tasks in both mood conditions.

Such a qualitative dissociation between the effects of an inter-group manipulation on the expression of learning has already been reported in previous studies. For example, Destrebecqz and Cleeremans [46] observed that two groups differing by their opportunity to prepare for the next stimulus in a SRT task both displayed sequential knowledge through their RT performance, but differed regarding the extent to which knowledge can be projected onto performance in a direct task. Similarly, numerous studies comparing performance in intentional (i.e., instructed to search for regularities) and incidental participants (i.e., who were given neutral instructions that did not refer to a systematic pattern), and who clearly differ regarding their explicit knowledge and processing strategy, reported similar direct and indirect measures of learning in both groups [39], [47], [48].

Interestingly, the fact that being in a sad mood during the acquisition of some knowledge would lead to a subsequent more conscious access to this knowledge might explain, at least partly, why negative stimuli are usually better recalled than neutral ones (e.g., [49]–[50]). Indeed, the higher recall rates associated with sad words as compared with neutral words (which did not induce any mood) might be attributed to the transient sad mood induced by their initial presentation. Further studies should systematically investigate the possibility that negative words induce an analytic processing style promoting conscious processing and, consequently, higher levels of performance in recall.

Although the interaction between Mood and Delay only approached significance, it is interesting to note that, even though they did not perform better, ‘sad’ participants were more confident in their responses than neutral ones when the learning tasks immediately followed the Exposure/Mood induction phase. This may be related to the notion of ‘depressive realism’, namely that depressed people are more realistic and accurate than non-depressed, control people (e.g., [51]). As controls are generally considered as unrealistically positive and over-confident, the depressive realism hypothesis would predict higher confidence in control than depressed participants. However, as Fu, Koutstaal, Fu, Poon, and Cleare [52] recently suggested, in situations where controls are under-confident, the depressive realism hypothesis predicts that depressed, realistic participants will be more confident than controls. In the ‘Delay 0′ condition of our study, ‘neutral’ participants indeed provided more ‘guess’ responses when they performed correctly than sad participants did (74.681%, SD = 26.454 vs. 58.334%, SD = 31.162, F(1, 62) = 5.118, p = .027, Cohen’s d = .566), supporting that ‘neutral’ participants were under-confident.

However, there was no difference in confidence levels between ‘neutral’ and ‘sad’ participants when there was a break between both phases, due to ‘neutral’ participants being more confident than when there was no break. These changes in confidence levels are difficult to explain, but might be related to ‘neutral’ participants becoming more positive and less negative during the break, increasing their confidence. Further studies should address this point specifically.

One limitation of the present study is that we did not control how much attention participants devoted to the stories. Participants in the sad group may have focused on the shapes to the detriment of the story. Indeed, alternative, non-exclusive explanations for higher awareness in sad participants may be related to the sad content of the story. On the one hand, it may be the case that participants avoid to immerse in an unpleasant story and rather engage attention in the visual items. Previous studies indeed showed that negative stimuli might elicit avoidance [53], [54], probably reflecting avoidance of the stress inductor agent [55]. Remarkably, this would not prevent the MIP to be effective. On the other hand, a sad mood may increase motivation to engage in unrelated cognitive tasks (e.g., [13]). Hence, this attentional engagement towards the shapes at the detriment of the story may have favored the acquisition of conscious knowledge about the sequences. To disentangle between these interpretations and to control for any trade-off between listening to the story and paying attention to the sequences, further studies should also measure memory for the story.

### Conclusions

To our best knowledge, the present study is the first to investigate the influence of a sad vs. neutral mood on implicit and explicit statistical learning. We showed that, although mood induction did not influence direct and indirect measures of learning, the analytic processing peculiar to negative affects elicited increased conscious access to the acquired knowledge. These results support the notion that individual differences impact on explicit but not on implicit learning, in line with Reber’s robustness principle.
